# Phytochemistry, Bioactivity, and Ethnopharmacology of the Genus *Lepechinia* Willd. (Lamiaceae): A Review

**DOI:** 10.3390/plants13040481

**Published:** 2024-02-08

**Authors:** Jorge Ramírez, Gianluca Gilardoni, Matteo Radice, Vladimir Morocho

**Affiliations:** 1 Departamento de Química, Universidad Técnica Particular de Loja, Loja 1101608, Ecuador; ggilardoni@utpl.edu.ec (G.G.); svmorocho@utpl.edu.ec (V.M.); 2 Department of Biosciences, Biotechnology and Environment, University of Bari Aldo Moro, Via E. Orabona, 4, 70125 Bari, Italy; matteo.radice@uniba.it

**Keywords:** *Lepechinia*, guajanes, aromadendranes, eudesmanes, cadinanes, abietanes

## Abstract

The genus *Lepechinia* (Lamiaceae) involves several aromatic shrubs that are distributed only in the American continent, inhabiting mountain areas, mainly in the Andean region of South America. Based on the PRISMA approach, we selected and critically analyzed 48 research articles. From a phytochemical point of view, most of the secondary metabolites reported in *Lepechinia* spp. are terpenes and terpenoids, with a few exceptions comprising flavonoids and other shikimic acid derivatives. On the one hand, sesquiterpenoids of the guajane, aromadendrane, eudesmane, and cadinane groups are characteristic of essential oils, together with (*E*)-β-caryophyllene as the main representative of its chemical family. On the other hand, abietane diterpenoids are the prevalent compounds described in non-volatile fractions. Many biological activities and traditional medical uses have been reported for both pure metabolites and complex mixtures (e.g., essential oils). Regarding ethno-medical uses, the treatment of muscle pain, headache, toothache, diabetes mellitus, uterine tumors, uterine infections, and diarrhea has been reported. Concerning their verified biological activities, insecticidal, antifungal, antioxidant, and anticholinesterase properties have been described. Furthermore, some data concerning anti-herpetic activity have been reported.

## 1. Introduction

Biodiversity has, for a long time, been the main source of pharmaceutical products, through the use of medicinal plants. Today, natural products still sometimes provide inspiration to chemists regarding the design of new pharmaceutical active principles, and, in many countries, vegetal drugs constitute the main source of traditional medicines [1]. 

Lamiaceae, one of the most important herbal families, incorporates a wide variety of plants, most of them characterized by biological and medical applications. This family comprises 224 genera and more than 5600 species distributed across the world. The best-known members of this family belong to a group of medicinal and aromatic plants, including the genus *Lepechinia* [2].

The genus *Lepechinia* Willd consists of 43 species that grow from Northern California in the western USA to central Argentina in South America [3,4,5,6]. *Lepechinia* spp. range from perennial herbs to shrubs, are rarely gynodioecious or dioecious, and are often aromatic; their leaves range from entire to toothed and are often rugose; and they have terminal and often axillary inflorescence [4,7]. A detailed bibliographic study was performed, including revision of papers from the years 1948 to 2023 regarding the genus *Lepechinia.* In this research, 64 plant species were mentioned, according to the botanical literature [8]. Focusing on these data, the name of genera and all the scientific names of the species were selected.

The goal of this review is to provide information on the ethnomedical uses, phytochemistry, and biological activities of different *Lepechinia* spp.

## 2. Research Strategies and Literature Sources

**Figure 1:**
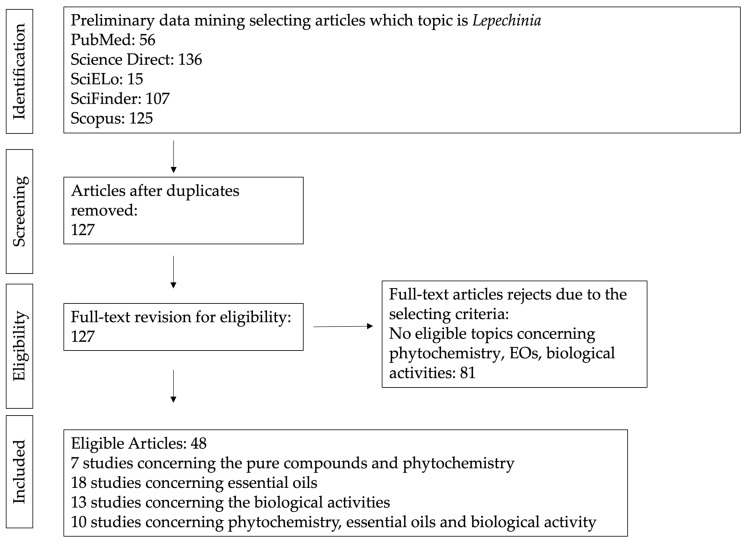
Flowchart for the search process and selection of the studies considered for the review.

The present review was developed according to the PRISMA guidelines [9]. The data included in this paper have been retrieved using the keywords, “*Lepechinia* medicinal plants”, “*Lepechinia* phytochemistry”, “*Lepechinia* ethomedicine uses”, “*Lepechinia* essential oils”, and “*Lepechinia* biological studies” in the following electronic databases: PubMed (https://pubmed.ncbi.nlm.nih.gov/, accessed on 30 September 2023) ScienceDirect (https://www.sciencedirect.com/, accessed on 30 September 2023), SciFinder (https://scifinder.cas.org/, accessed on 30 September 2023), SciELO (https://scielo.org/, accessed on 30 September 2023), and Scopus (https://www.scopus.com/, accessed on 30 September 2023). In order to manage all the bibliographic references, Mendeley Desktop software version 1.19.8 was used. Three reviewers extracted data independently, avoiding the duplication of data, and they searched for and selected all key words individually. The process of datamining is synthetized in the flowchart in Figure 1. When a scientific paper could not be found online, we asked the authors to send it to us. For the final step of the study, articles in English and Spanish were selected, together with data from patents. Congress abstracts and symposiums were excluded because they were considered not sufficiently complete to warrant an exhaustive comparison with full articles.

## 3. Results and Discussion

### 3.1. Compounds Isolated from Lepechinia spp. through Solvent Extraction and Their Biological Activities

**Figure 2:**
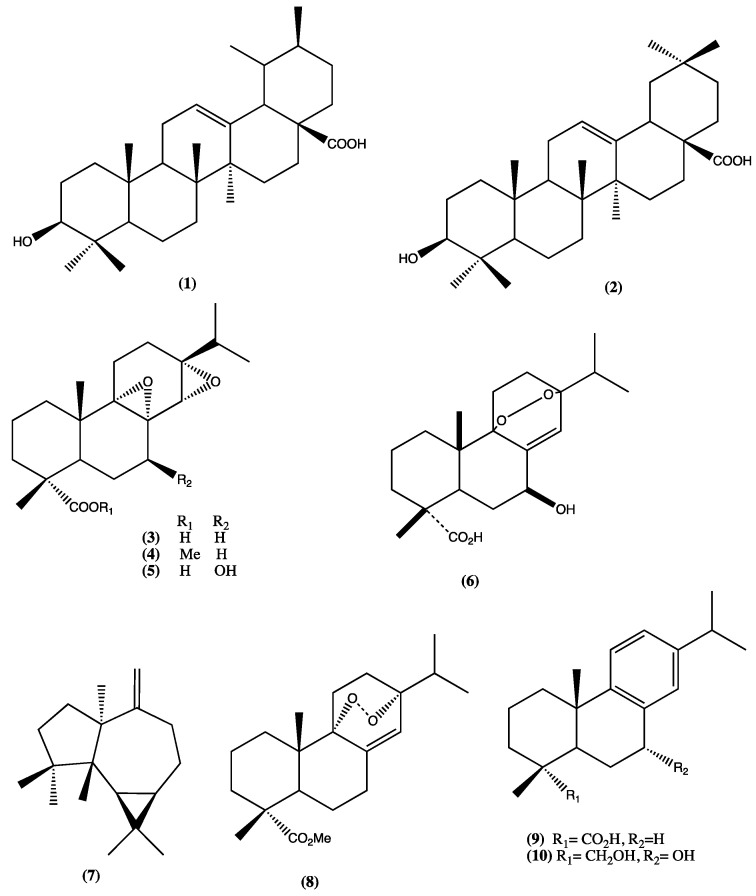
Compounds isolated from *L. caulescens.*

Phytochemical investigation of *Lepechinia* spp. has shown the presence of tricyclic diterpenes, flavonoids, pentacyclic triterpenes, etc. [5,6]. Two know pentacyclic triterpenes, ursolic acid (**1**) and oleanolic acid (**2**), a new diterpene (**3**), its methyl ester (**4**), and one natural endoperoxide (**5**), have been isolated from *L. caulescens*. In another research work, 7β-Hydroxy-abieta-8(14)-en-18-oic 9α,13α-endoperoxide (**6**) was also isolated from the same plant [6,10,11]. Compounds **1** and **2** produced a significant vasodilator effect in a concentration-dependent and endothelium dependent-manner [12]. Ursolic acid is probably the most extensively studied molecule among those mentioned above, with potential applications of ursolic acid and its derivatives as anti-tumour agents; there are also several patents filed [13,14,15,16]. Finally, recent studies performed by Al-kuraishy et al. showed a preliminary result concerning the control of hyperinflammation and oxidative stress in SARS-CoV-2 [17]. Spathulenol (**7**), 9α,13α-epidioxyabiet-8(14)-en-18-oic acid methyl ester (**8**), dehydroabietic acid (**9**), and 9β-hydroxydehydroabietyl alcohol (**10**) (Figure 2, compounds **1**–**10**) were also isolated from *L. caulescens* [10] and showed spasmolytic activity.

**Figure 3:**
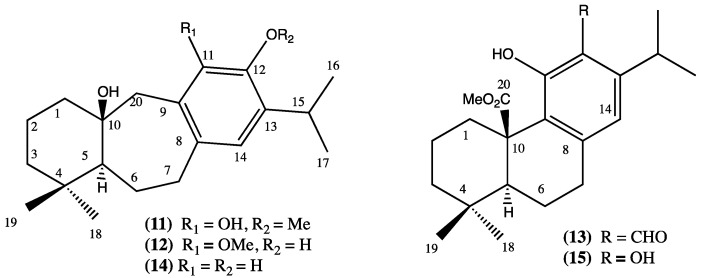
Compounds isolated from *L. mayenii* and *L. hastata.*

From the aerial parts of *L. meyeni*, the abietane diterpenes salvicanol (**11**), isosalvicanol (**12**), 12-formyl-11-hydroxy-abieta-8,11,13-trien-2-oic acid methylester (**13**), pisiferanol (**14**), and carnosic acid methyl ester (**15**) (Figure 3, compounds **11**–**15**) have been isolated. Carnosic acid is the main diterpenoid in *L. hastata* [18].

**Figure 4:**
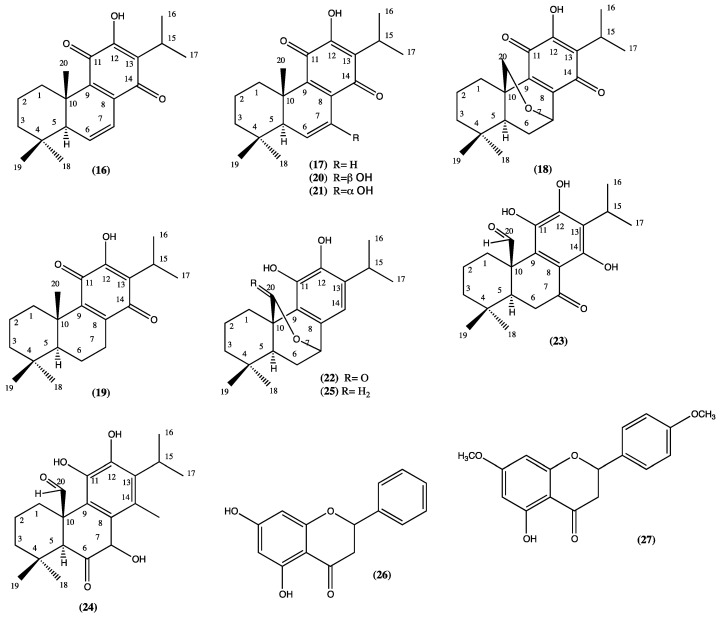
Compounds isolated from *S. chamaedryoides.*

**Figure 5:**
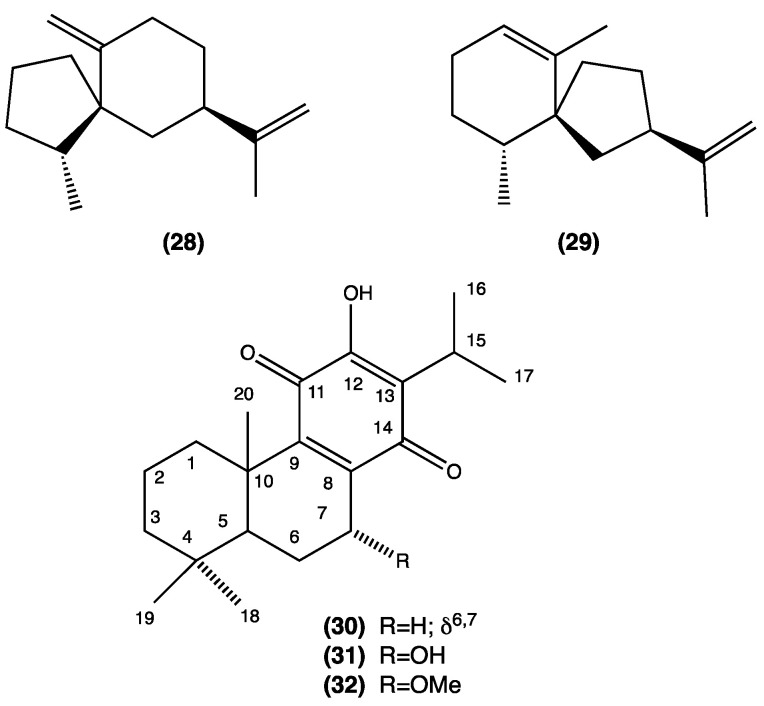
Compounds isolated from *L. bullata.*

From the aerial parts of *Sphacele chamaedryoides*, (synonym *L. chamaedryoides*) [19], ten abietane diterpenes have been isolated: 6,7-dehydroroyleanone (**16**), royleanone (**17**), 7,20-epoxyroyleanone (**18**), ferruginol (**19**), taxoquinone (**20**), horminone (**21**), carnosol (**22**), 7-oxo-11,12,14-trihydroxy-abieta-8,11,13-trien-20-al (**23**), 7-oxo-7a,11,12-trihydroxy-abieta-8,11,13-trien-20-al (**24**), deoxocarnosol (**25**), sphatulenol (**7**), pinocembrin (**26**), and 5-hydroxy-4′,7-dimethoxyflavone (**27**) [20] (Figure 4, compounds **16**–**27**). Several compounds present in this plant showed a higher gastroprotective effect than lansoprazole, and the cytotoxic effect of most compounds was measured at fairly high concentrations and lacked cell specificity; instead, compounds **16** and **20** showed selective cytotoxicity against AGS cells and fibroblasts, respectively [20].

(-)-Spirolepechinene (**28**) is a new spirosesquiterpene that was isolated from *L. bullata* together with a known sesquiterpene, (-)-premnaspirodiene (**29**) [21]. From the MeOH extract of *L. bullata*, three cytotoxic diterpene quinones were also isolated: 6,7-dehydroroyleanone (**30**), horminone (**31**), and the new compound 7-*O*-methylhorminone (**32**). Compound **31** inhibited the growth of *Trypanosoma cruzi* [22] (Figure 5, compounds **28**–**32**).

**Figure 6:**
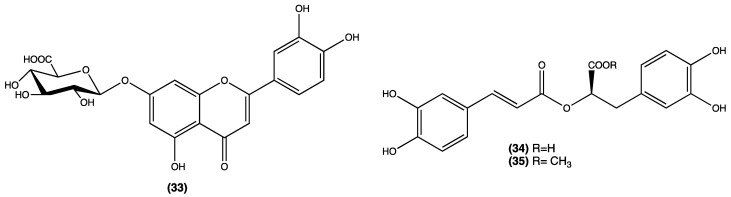
Compounds isolated from *L. graveolens.*

From *L. graveolens*, collected in Mexico, three antioxidative phenolic compounds have been isolated, namely, luteolin-7-*O*-glucuronide (**33**), rosmarinic acid (**34**), and rosmarinic acid methylester (**35**) (Figure 6, compounds **33**–**35**) [23]. Rosmarinic acid is undoubtedly the most relevant compound and has been extensively studied for its antimicrobial and antioxidant effects for use in anti-diabetic and potentially dietary supplements in cancer treatment [24,25,26,27].

**Figure 7:**
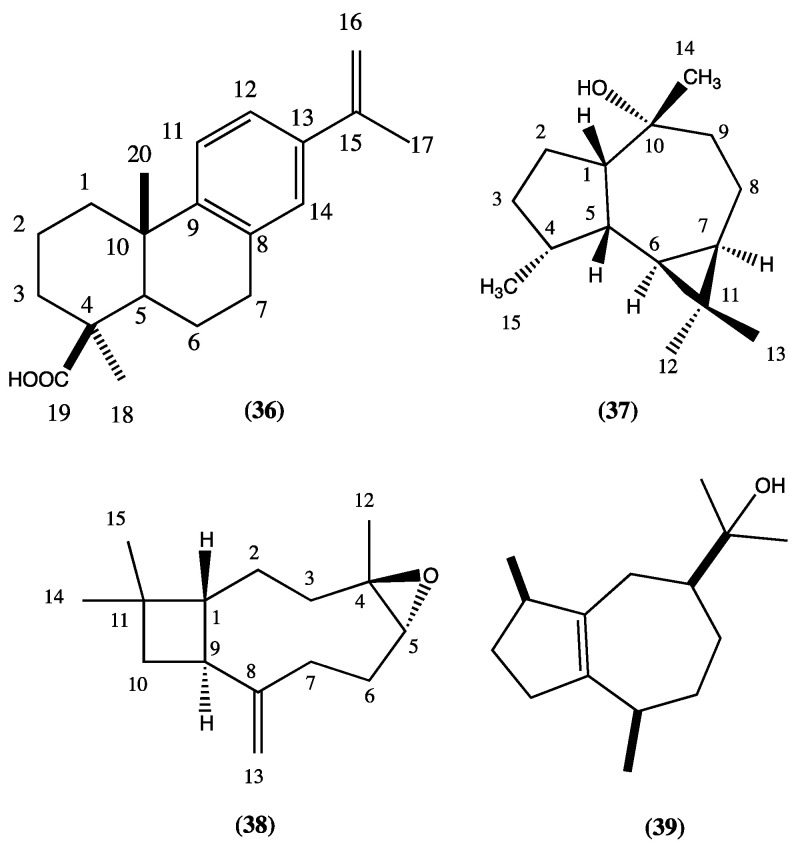
Compounds isolated from *L. radula* and *L. paniculata.*

Spathulenol (**7**), angustanoic acid E (**36**), and 5-hydroxy-4′,7-dimethoxyflavone (**27**) were isolated from *L. radula*; (-)-ledol (**37**), (-)-caryophyllene oxide (**38**), guaiol (**39**), and carnosol (**22**) were found in *L. paniculata* (Figure 7 compounds **36**–**39**) [28].

**Figure 8:**
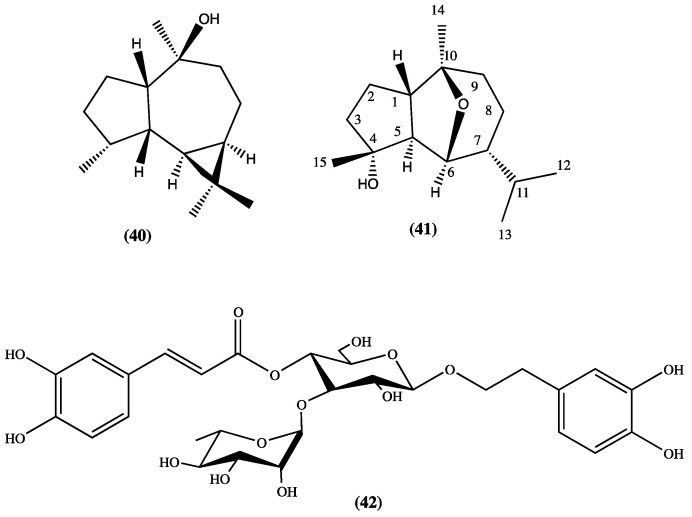
Compounds isolated from *L. mutica* and *L. speciosa*.

From *L. mutica* (Benth.), an endemic plant from Ecuador, the following compounds have been isolated and identified: carnosol (**22**), viridiflorol (**40**), ursolic acid (**1**), oleanolic acid (**2**), chrysothol (**41**), and 5-hydroxy-4′,7-dimethoxyflavone (**27**) [29]. Carnosol seems to be active against the “blast disease” caused by the fungus *Pyricularia oryzae*; furthermore, it has shown a promising selective inhibitory activity against butyrylcholinesterase [29,30]. Verbascoside (**42**) has been isolated form *L. speciosa* [31] (Figure 8, compounds **40**–**42**).

### 3.2. Composition and Biological Activities of Essential Oils from Genus Lepechinia 

**Figure 9:**
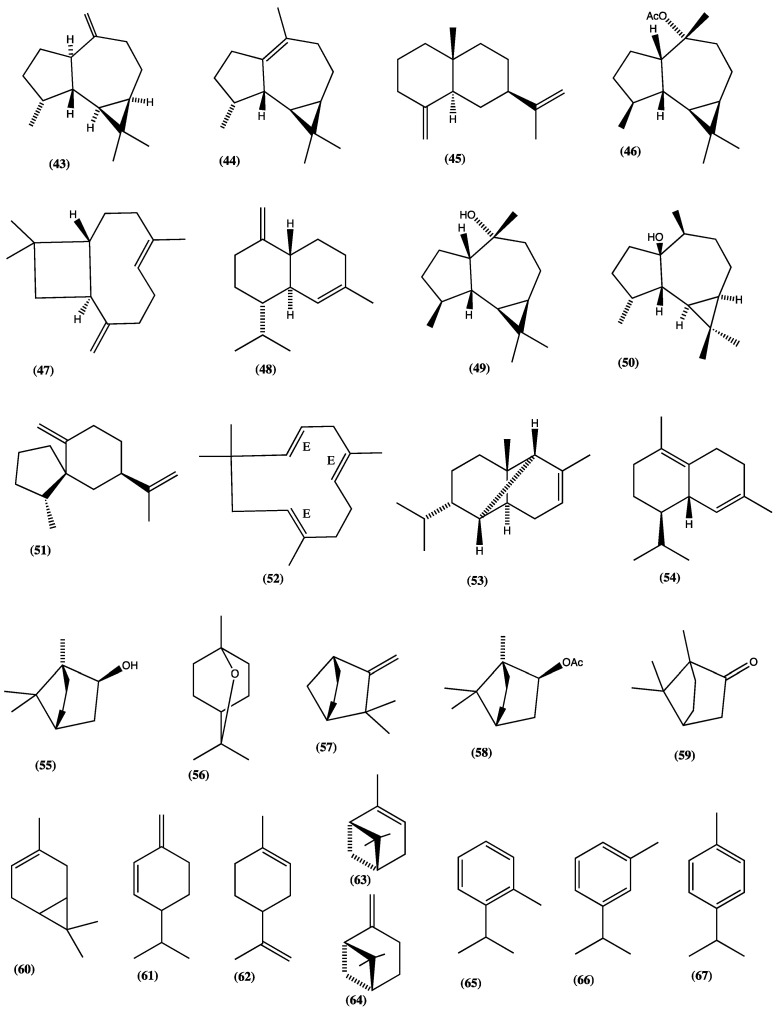
Compounds identified in the essential oils of *Lepechinia* spp.

Sesquiterpenes, diterpenes, triterpenes, and flavonoids have been isolated from different species of this genus. Some species are used for their anti-tumoral and insulin-mimetic properties, to treat uterine infections, or to calm stomach pains [32]. Regarding the volatile essential oil components, 18 species of the genus *Lepechinia* have been studied so far; they are: *L. bullata* [21], *L. betonicifolia* [33,34], *L. calycina* [35], *L. caulescens* [36], *L. conferta* [32], *L. chamaedryoides* [37,38], *L. floribunda* [39,40,41,42], *L. graveolens* [41], *L. heteromorfa* [28], *L. meyeni* [41,43,44], *L. mutica* [45,46,47], *L. paniculata* [48,49], *L. radula* [50], *L. salviaefolia* [51], *L. salvifolia* [52], *L. schiedeana* [53,54,55], *L. urbanii* [56], and *L. vulcanicola* [52]. Given the heterogeneity of the compounds identified in the *Lepechinia* spp., it is not possible to establish a characteristic pattern of compounds for the genus. In Ecuador, the essential oils of four species belonging to the genus *Lepechinia* have been studied [28,45,46,47,48,49,50]. In Table 1, we have reported the main volatile compositions and biological activities from *Lepechinia* spp., and, in Figure 9, the structures of the main compounds identified in their essential oils are shown: armomadendrene (**43**), viridiflorene (**44**) β-selinene (**45**), ledyl acetate (**46**), β-caryophyllene (**47**), g-cadinene (**48**), ledol (**49**), (-)-palustrol (**50**), (-)-spirolepechinene (**51**), α-humulene (**52**), α-copaene (**53**), d-cadinene (**54**), borneol (**55**), 1,8-cineole (**56**), camphene (**57**), bornyl acetate (**58**), camphor (**59**), d-3-carene (**60**), β-phellandrene (**61**), limonene (**62**), α-pinene (**63**), β-pinene (**64**), *o*-cymene (**65**), *m*-cymene (**66**), and *p*-cymene (**67**).

The biological activity of an essential oil is mainly due to the synergistic effect of its compounds, so it is difficult to attribute the effect of in vivo and in vitro tests to a single molecule or predominant compound. However, some of the molecules listed a few times in Table 1 are common in various essential oils and have been the subjects of several studies. Limonene is a monocyclic monoterpene that may be extracted abundantly from citrus peel waste or obtained via microbial biosynthesis [57,58]; this compounds and its derivatives are present as common additives in several markets, such as cosmetics, food, pharmaceuticals, cosmetics, and biobased polymers [57,59,60]. 1,8-cineole (eucalyptol) is another of the most investigated compounds in recent years, and various pieces of evidence point to its potential as a phytochemical treatment for respiratory disorders, such as chronic obstructive pulmonary disease (COPD), due to its mucolytic, anti-inflammatory, antimicrobial, bronchodilator, and antioxidant activities [61,62,63].

### 3.3. Ethnopharmacology and Traditional Uses for the Genus Lepechinia 

Several *Lepechinia* spp. are valued in the horticultural trade, and North and South American indigenous groups commonly use *Lepechinia* plants for medicinal purposes, such as the treatment of muscle pain, headache, and toothache [45], diabetes mellitus, uterine tumors, uterine infections, and diarrhea [5,31]. *L. caulescens* is the most mentioned species concerning folk medicine, and its traditional use has been reported in cases of stomach ailments, hypertension, diabetes, dysmenorrhea, and gastrointestinal infections. The aqueous infusion, obtained via decoction from the whole plant, is the most common traditional beverage [6,10,39,55,56]. An extract from *L. caulescens* has been patented as a cosmetic agent [64,65]. Also, the teas of *L. graveolens* and *L. hastata* (root extract) have been mentioned in the treatment of stomach ailments [20] and uterine infections, respectively [65,66]. The decoction of the leaves of *L. meyenii* is used as an antispasmodic, digestive, and carminative agent and for the treatment of coughs and diarrhea [66,67]. Regarding *L. schiediana*, two studies have cited the use of it in tea in folk medicine as a diuretic, as a remedy for kidney infections and stomach illness, and for the treatment of skin irritation [43,68]. The leaves of *L. mutica* are used to treat headaches, nervous affections, and startle disease [45,46,69]. The leaves of *L. radula* are used to treat “mal de aire” and aches in muscles and bones [50,69]. Regarding *L. paniculata*, it is used to treat headaches via the direct application of its buds to the head; its flowers are used for the treatment of nervous system affection and for evil air “mal aire’ and “espanto” [48,49]. In Table 2, there are more details about the ethnopharmacology and traditional uses of some *Lepechinia* spp.

### 3.4. Geography and Aim of the Studies

**Figure 10:**
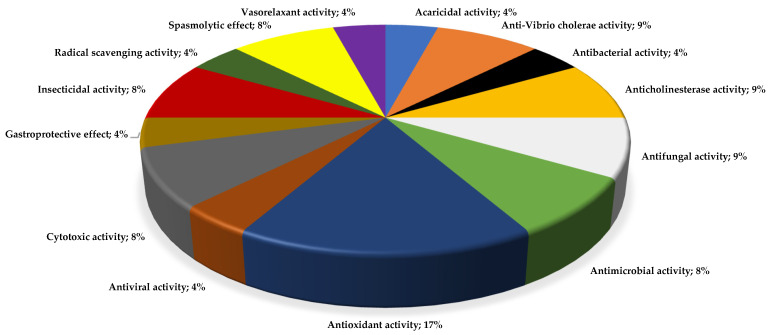
Diversity of biological activity in *Lepechinia* spp. The percentages refer to the corresponding articles compared to the total number of biological activity reports.

Using these criteria [9], we were able to collect 48 papers, for which 52 different studies had been carried out on 20 species of the *Lepechinia* genus (Table 3) in relation to essential oils, phytochemistry, and different biological activities, such as: insecticide-repellent activity, anticholinesterase activity, spasmolytic effects, vasorelaxant activity, anti-vibrio cholerae activity, gastroprotective effects, cytotoxicity activity, antibacterial activity, antifungal activity, and antioxidant activity, among other. Most species of the genus *Lepechinia* have been investigated for their antioxidant activity (see Table 3 and Figure 10). On the one hand, the predominance of antioxidant activity can be explained by the relatively easiness of these assays and the fact that practically all vegetal species contain important amounts of polyphenolic compounds, typically characterized by this property. On the other hand, almost 10% of all the reported activities corresponded to the cholinergic capacity of essential oils or purified metabolites. Most of these studies were conducted in Ecuador.

**Figure 11:**
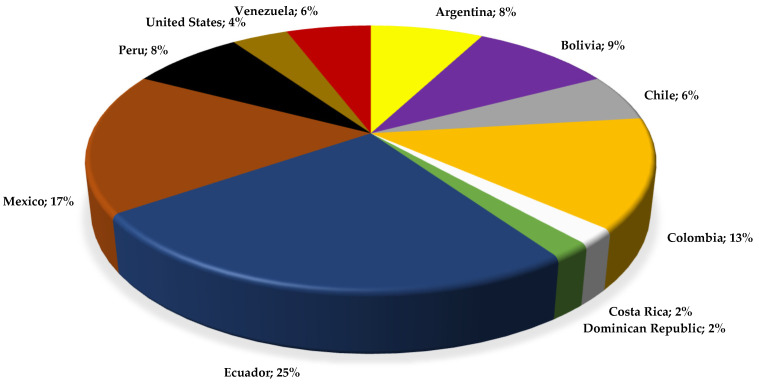
Geographical origin of studies in *Lepechinia* spp. Percentages refer to the corresponding articles compared to the total number of geographical origin reports.

The studies have been distributed mainly in Ecuador (13), Mexico (9), Colombia (7), and Bolivia (5), with several in Argentina (4), Peru (4), Chile (3), Venezuela (3), the United States (2), Costa Rica, and the Dominican Republic (1); see Figure 11. About one quarter of the total phytochemical studies on the genus *Lepechinia* were carried out in Ecuador, where an important number of endemic and native species have attracted relevant academic interest over the last ten years. This interest coincides with a period of development in the scientific research on all fields of biodiversity in Ecuador.

## 4. Conclusions

This review demonstrates the great potential of the genus *Lepechinia* as a source of interesting secondary metabolites, often characterized by relevant biological activities. Almost all the compounds described in *Lepechinia* spp. were terpenes and terpenoids, with few exceptions among flavonoids and other shikimic acid derivatives. On the one hand, sesquiterpenoids of the guajane, aromadendrane, eudesmane, and cadinane families dominated the essential oils, together with (*E*)-β-caryophyllene as the main representative of its group. On the other hand, non-volatile fractions were characterized by diterpenoids of the abietane family. A wide spectrum of biological activities and traditional medical uses were reported for both pure metabolites and complex mixtures (e.g., essential oils). 

## Figures and Tables

**Table 1:** Main compounds and biological activities in essential oils from *Lepechinia* spp.

*Lepechinia* spp.	Place of Collection	Essential Oil	Main Compounds (>5%)	Biological Activities
*L. bullata*	Santo Domingo (Estate of Mérida, Venezuela)	Leaves (hydrodistillation)	(-)-spirolepechinene (20%), (-)-premnaspirodiene (45%) [21]	-
*L. betonicifolia*	Bucaramanga (Colombia) and Loja (Ecuador)	Aerial parts (Microwave-assisted hydrodistillation)	Limonene (27.5%), a-pinene (19.4%), β-pinene (9.5%), and trans-β-caryophyllene (6.8%) [33] β-pinene (30.45%), sabinene (27.98%), α-pinene (4.97%), β-phellandrene (4.79%), E-caryophyllene (4.44%), and limonene (3.84%) [34]	Registers repellency percentages > 90% after 2 and 4 h against *Tribolium castaneum* [33] EO exerted a strong inhibitory effect over the AChE enzyme, with an IC_50_ value of 74.97 ± 1.17 lg/mL [34]
*L. calycina*	California (North America), University of California Botanical Gardens	-	1,8-cineole (19.7%), camphor (17.5%), d-3-carene (17.4%), camphene (7.8%), α-pinene (6.5%), and caryophyllene (5.7%) [35]	-
*L. caulescens*	Michoacán (México)	Aerial parts	Borneol (16.38%), camphor (15.16%), trans-caryophyllene (15.14%), spathulenol (8.51%), and aromadendrene (8.01%) [36]	Active against some strains of *Vibrio cholerae* [36]
*L. conferta*	Páramo of La Negra (Venezuela)	Leaves and flowers (hydrodistillation)	The concentration of sesquiterpenes is slightly higher in the flowers (57.5%) than in the leaves (53.5%); the most abundant constituents are the same in both: ledol (28.9% and 24.2%), δ3-carene (10.5% and 11.0%), β-phellandrene (11.7% and 9.7%), and aromadendrene (3.6% and 4.5%), respectively [32]	-
*L. chamaedryoides*	Tomé (Concepción, Chile)	Aerial parts (hydrodistillation)	α-phellandrene (13.0%), β-caryophyllene (10.3%), T-cadinol (10.4%), spathulenol (6.4%), limonene (6.0%), and g-cadinene (5.9%) [37]	Insecticidal activity on Diptera larvae, *Drosophila melanogaster* Meigen [36]
*L. floribunda*	Cordoba, San Luis, and Buenos Aires Provinces (Argentina) [39,40,42] and Mizque (Bolivia) [41]	Aerial parts (hydrodistillation)	1,8-cineole (27.5%), camphene (16.6%), and camphor (12.9%) for *L. floribunda* from Argentina [32,39,40]. Bornyl acetate (11–12%), β-caryophyllene (9–10%), and camphene (5.7%) for *L. floribunda* from Bolivia [41]	Anti-quorum sensing (QS) [39] and antimicrobial activity against *Paenibacillus larvae* [40]
*L. graveolens*	Cochabamba region of Chapare (Inkachaca-Bolivia)	Aerial parts (hydrodistillation)	β-caryophyllene (22.1%), d-cadinene (6.1%), α-humulene (5.6%), β-phellandrene (5.3%), and g-cadinene (5.3%) [41]	-
*L. heteromorfa*	Saruguro, Province of Loja (Ecuador)	Leaves (hydrodistillation)	Viridiflorene (27.3%), (-)-Ledol (21.2%), Spirolepechinene and (E)-β-caryophyllene (7.1% each), and allo-aromadendrene (6.1%) [28]	-
*L. meyenii*	Arani (Cochabamba, Bolivia) [41,43] San Martín de Porras (Perú) [44]	Leaves (hydrodistillation)	β-pinene (12.6%) and limonene (~5%) [43]. β-Pinene (12.6%), α-pinene (9.7%), and d-3-carene (6.9%) were the main constituents of the A oil, whereas α-pinene (25.0%), β-pinene (9.2%), and limonene (8.3%) were the most important products in the B oil. In both cases, tau-cadinol and epi-α-bisabolol (~9–12% each) were the main constituents; g-cadinene (~5–6%) was the main constituent in both oils [41]. α-Pinene (29.87%), Eucalyptol (13.25%), and β-Pinene (9.64%) [43]	Antioxidant activity [44]
*L. mutica*	Cerro el Villonaco (Loja, Ecuador) [45] Quilanga Region in Loja Province, Ecuador [29,46,47]	Leaves and flowers (hydrodistillation)	β-phellandrene (30%), camphene (13%), limonene (8%), 3-carene (6%), α-pinene (6%), and isocaryophyllene (5%) [44]. Shyobunol (10.80%), D^3^-carene (8.69%), d-cadinene (6.96%), and globulol (5.91%) [46,47] δ-3-carene (24.2%), eudesm-7(11)-en-4-ol (13.0%), thujopsan-2-α-ol (11.9%), β-pinene (8.0%), and valerianol (5.2%) for the flower essential oil [29]	Moderate in vitro activity against five fungal strains, especially against *M. canis*, a causal agent for pet and human infections [46]. Promissory acaricidal activity against larvae and engorged adult females of the common cattle tick, *Rhipicephalus microplus* [47]
*L. paniculata*	Barrio Acacana of San Lucas (Loja, Ecuador) [48]. El Tablon (Loja, Ecuador) [49]	Leaves and flowers (hydrodistillation and steam distillation)	Aromadendrene (24.6%), viridiflorene (12.4%), β-selinene (7.4%), valencene (6.7%), and β-phellandrene (7.7%) [48]. 1,8-Cineole (18.73%), β-Pinene (16.27%), δ-3-Carene (12.44%), α-Pinene (11.10%), (E)-Caryophyllene (9.88%), β-Phellandrene (8.62%), and Guaiol (8.58%) [49]	Moderate inhibitory activity against anticholinesterase activity, with IC_50_ values of 38.2 ± 2.9 mg/mL against AChE and 47.4 ± 2.3 mg/mL against BuChE, whereas in the EO of the flowers, the inhibitory activity was much more marked, with IC_50_ values of 28.2 ± 1.8 µg/mL against AChE and 28.8 ± 1.5 µg/mL against BuChE [49]
*L. radula*	Guachanamá (Loja, Ecuador)	Aerial parts (hydrodistillation)	δ-3-carene (19.9%), β-pinene (17.0%), (E)-β-caryophyllene (9.7%), and (E-E)-α-farnesene (9.4%) [50]	Strong antifungal activity against *Trichophyton rubrum* and *Trichophyton mentagrophytes* [50]
*L. salviaefolia*	Mérida, Venezuela	Leaves (hydrodistillation)	The major constituents of the palustrol-type (28 samples) were (-)-palustrol (19.1%), β-phellandrene (13.8%), borneol (11.8%), and camphene (7.2%). The oil of the premnaspirodiene-type (27 samples) was dominated by β-phellandrene (13.3%), borneol (12.3%), (-)-premnaspirodiene (9.4%), and cainphene (8.5%). The presence of a third chemotype (5 samples) with d-3-carene (12.9%), T-cadinol (9.1%), and borneol (8.4%) [51]	-
*L. salvifolia*	Sogamoso, Boyacá, and Bogotá D.C., Cundinamarca, Colombia	Whole plant (microwave-radiation-assisted hydrodistillation (MWHD))	Camphor (10.3%), Limonene (9.7%), *p*-Mentha-1(7),8-diene (7.4%), α-Pinene (6.9%), γ-Terpinene (6.7%), Camphene (5.9%), β-Pinene (5.3%), and trans-Caryophyllene (5.1%) [52]	Moderate antiviral activity against human herpes viruses (HHV-1 and HHV-2) at the concentration of 100 mg/mL [52]
*L. schiedeana*	Herbario Nacional Colombiano, Universidad Nacional de Colombia, Bogotá, Colombia	Whole plant (steam distillation (SD), simultaneous steam distillation–solvent extraction (SDE), supercritical fluid extraction (SFE) and microwave-assisted solvent extraction (MWE)) [52]	Ledol (36.9% for SD), d-3-Carene (22.0% for SDE), β-Pinene (8.04% for SDE), α-Terpinene (6.1% for SDE), and Myrcene (~5% for SD) [53]. In other research: Δ^3^-carene (21.4%), ledol (16.3%), β-pinene (11.3%), β-phellandrene (11.1%), and γ-terpinene (9.5%) [53]	Exhibited in vitro antioxidant activity [53,54]. Antioxidants BHA, vitamin E, and Trolox. The essential oils had a stronger protective effect against lipid peroxidation than BHA, vitamin E, and Trolox within the range of concentrations examined (1–20 g L^–1^) [55]
*L. urbanii*	La Vega, Dominican Republic	Leaves (steam distillation)	d-car-3-ene (32.55%), α-copaene (13.82), and d- cadinene (12.51) [56]	-
*L. vulcanicola*	Bogotá D.C., Cundinamarca, Colombia	Whole plant (microwave-radiation-assisted hydrodistillation (MWHD))	Limonene (18.9%), Germacrene D (10.4%), 1-Octen-3-ol (8.8%), *trans*-β-Caryophyllene (8.7%), α-Pinene (8.2%), and Bicyclogermacrene (5%) [52]	Anti-herpetic activity, with *Rf* values of 1 × 10^2^ and 1 × 10^3^ against one Tissue Culture Infectious Dose 50 (TCID_50_) of HHV-1 and HHV-2 [52]

**Table 2:** Ethnopharmacology and traditional uses of *Lepechinia* spp.

Scientific Name	Common Name	Ethnopharmacology and Traditional Uses
*L. caulescens*	Commonly known as “Bretónica” [70]	Different morphological structures and preparations are used for gastrointestinal ailments, diarrhea, and hypoglycemia [36]; for vomiting, diabetes, hypertension, and related diseases [71,72,73]; for dysmenorrhea and as an abortifacient [10]; and for stomach ailments [6]
*L. chamaedryoides*	Known as “Alhuelahuén” or “Male sage”	Emmenagogue and anti-inflammatory properties as an infusion [38]
*L. floribunda*	Local name: “Salvia morada” [42]	Antiseptic properties [74]
*L. graveolens*	White sage	As an infusion for the treatment of stomach ailments [23]
*L. hastata*	Locally known as “Chicura de la Sierra”, “Lavanda”, and “Lengua de buey”	Uterine infections as a root decoction [73]
*L. meyeni*	“Pampa salvia”, “Saluya” [41]	Treatment of coughs and diarrhea, antispasmodic [66]; digestive and carminative [67]
*L. mutica*	“Shalshon” in Kichwa or “Casa casa” in Spanish	“Espanto” (startle) [72]
*L. paniculata*	“Yayllon” or “Llanllum” in Kichwa	“Mal de aire”, a sort of evil eye, and against headache; flower infusions are used to treat nervous diseases [72]
*L. radula*	“Shalshon” or “Zhalshon” in Kichwa	The leaves are used to treat “mal de aire” and aches in muscles and bones [72]
*L. schiedeana*	“Salvia negra”	Skin irritations, muscle fatigue, and as a diuretic [53]; decoction used as a remedy for stomachache and kidney infections [71]

**Table 3:** Geographic origin and type studies conducted in *Lepechinia* spp., including phytochemical works.

Country	*Lepechinia* spp.	Part of Plant	Extraction	Type of Study	Reference
Colombia, Ecuador	*L. betonicifolia*	Aerial parts	Hydrodistillation, microwave-assisted hydrodistillation (MWHD)	Essential oil and biological activity (insecticide repellent, anticholinesterase activity)	[33,34]
Colombia, Venezuela	*L. bullata*	Leaves	Hydrodistillation, petroleum ether (PE) extract	Essential oil and phytochemistry	[21,22]
United States	*L. calycina*	Aerial parts	Hydrodistillation	Essential oil	[35]
Mexico	*L. caulescens*	Aerial parts, inflorescences, leaves	Acetone, dichloromethane, hexane, ethyl Acetate (EtOAc), and methanol (MeOH) extracts, hydrodistillation	phytochemistry, biological acivitity (spasmolytic effect, vasorelaxant activity, anti-vibrio cholerae activity)	[6,10,11,12,36,65,73]
Chile	*L. chamaedryoides*	Aerial parts, leaves	Hydrodistillation, petroleum ether (PE), and EtOAc extracts	Essential oil, biological acivitity (gastroprotective effect, cytotoxicity activity, and insecticidal activity)	[20,37,38]
Venezuela	*L. conferta*	Leaves, flowers	Hydrodistillation	Essential oil	[32]
Argentina, Bolivia	*L. floribunda*	Aerial parts, dry flowers, leaves	Hydrodistillation, steam distillation (SD)	Essential oil and biological activity (antimicrobial activity, antibacterial activity)	[39,40,41,42,74]
Bolivia	*L. graveolens*	Aerial parts, leaves	MeOH extract, steam distillation (SD)	Biological activity (antioxidant activity), essential oil, phytochemistry	[23,41]
Mexico, United States	*L. hastata*	Aerial parts	Acetone extract, petroleum ether (PE), chloroform and ethanol extracts	Phytochemistry, biological activity (antimicrobial activity)	[18,67,68]
Ecuador	*L. heteromorpha*	Aerial parts	Hydrodistillation	Essential oil	[28]
Bolivia, Peru	*L. meyenii*	Leaves, aerial parts	Acetone extract, steam distillation (SD), ethanol, MeOH and water extracts	Essential oil, phytochemistry, biological activity (antimicrobial activity, antioxidant activity), phenolic compounds)	[18,41,43,44,69,70]
Ecuador	*L. mutica*	Leaves and flowers	Ethyl Acetate (EtOAc) extract and hydrodistillation	Essential oil, phytochemistry, and biological activity (Antifungal activity, anticholinesterase activity, acaricidal activity)	[29,30,45,46,47,75]
Ecuador	*L. paniculata*	Aerial parts, leaves	Ethyl Acetate (EtOAc) extract, hydrodistillation	Essential oil, phytochemistry	[28,48,49]
Ecuador	*L. radula*	Aerial parts, leaves	Ethyl Acetate (EtOAc) extract, hydrodistillation	Essential oil, phytochemistry	[28,50]
Venezuela	*L. salviaefolia*	Leaves	Hydrodistillation	Essential oil	[51]
Colombia	*L. salvifolia*	Whole plants	Microwave-radiation-assisted hydrodistillation (MWHD)	Essential oil and biological activity (antiviral activity)	[52]
Colombia, Costa Rica	*L. schiedeana*	Aerial parts, leaves, flowers, whole plants	Hydrodistillation, steam distillation (SD)	Essential oil and biological activity (antiviral activity, antioxidant activity, radical scavenging activity)	[53,54,55,71]
Brazil	*L. speciosa*	Aerial parts	Ethanol extract	Phytochemistry and biological activity (antiviral and cytotoxic activity)	[5,31]
Dominican Republic	*L. urbanii*	Leaves	Steam distillation (SD)	Essential oil	[56]
Colombia	*L. vulcanicola*	Whole plants	Microwave-radiation-assisted hydrodistillation (MWHD)	Biological activity (antiviral activity)	[52]

## Data Availability

Not applicable.
